# The Cost Dynamics of Tuberculosis Comprehensive Programme: A Systematic Review

**DOI:** 10.21315/mjms-05-2025-377

**Published:** 2026-04-30

**Authors:** Sinta Dewi Lestyoningrum, Adistha Eka Noveyani, Wahyu Gito Putro, Wahyu Pudji Nugraheni, Debri Riski Faisal, Indah Pawitaningtyas, Muhammad Agus Mikrajab

**Affiliations:** 1 Research Center for Public Health and Nutrition, Research Organization for Health, National Research and Innovation Agency (BRIN), Bogor, West Java, Indonesia; 2 Department of Epidemiology, Faculty of Public Health, Universitas Jember, Jember, East Java, Indonesia; 3 Department of Public Health, Faculty of Medicine, Universitas Muhammadiyah Semarang, Semarang, Central Java, Indonesia

**Keywords:** communicable disease, cost of effectiveness, cost of programme, health economics, health programme, tuberculosis

## Abstract

Tuberculosis (TB), as a significant global health challenge, remains leading the increasing burden of its disease. There is an essential requirement to understand and manage the costs of the TB treatment programme to ensure the sustainability of the TB control programme. The aims of this study are to synthesise existing literature on the economic implications of TB treatment programmes, focusing on the cost of the programme associated with drug-susceptible and drug-resistant TB. Following Preferred Reporting Items for Systematic Reviews and Meta-Analyses (PRISMA) guidelines and utilising the Population, Intervention, Comparison, Outcomes, and Study Design (PICOS) framework, studies published from 2013 to 2024 were systematically searched from PubMed, Scopus, and ScienceDirect. Only economic evaluations were included, resulting in 11 articles that provided diverse regional insights into TB costs. The review highlights the considerable financial burden of TB’s management, with diverse results from the literature. This study reveals that the combined strategy of the TB programme increases the cost-effectiveness at USD9,050.00 per disability-adjusted life year (DALY) and USD443.62 per TB case. The highest cost of the TB programme was reported for the passive case finding (PCF) at USD226.13 for adults. The funding gap was reported at USD301.62 million from 2018 to 2022. Effective TB management requires some comprehensive financial strategies to mitigate the economic burden that affects the direct and indirect national expenditure. Focusing on combining the strategies of passive and active case finding, increasing the treatment’s accessibility, and supporting the drug innovation are believed to be the strategies to enhance the achievement of the TB programme, as well as increasing the cost-effectiveness of its programme.

## Introduction

Tuberculosis (TB) remains a significant global health challenge, with approximately 10.0 million new cases and 1.5 million deaths reported annually. Despite advances in treatment and diagnosis, TB remains a leading cause of morbidity and mortality, particularly in low-and middle-income countries where healthcare resources are often scarce. The economic burden of TB treatment is substantial, affecting not only healthcare systems but also patients and their families. The fight against TB has evolved significantly since the discovery of antibiotics in the mid-20th century. However, the emergence of drug-resistant strains, particularly multidrug-resistant TB (MDR-TB) and extensively drug-resistant TB (XDR-TB), has complicated treatment efforts. The World Health Organization (WHO) established the End TB Strategy, aiming to reduce TB incidence by 90% by 2035, which requires effective, cost-efficient management strategies (1).

Current challenges include drug-resistant TB, treatment regimens, healthcare system burden, and economic impact. The rise of MDR-TB, which is resistant to at least isoniazid and rifampicin, presents a formidable challenge to TB control efforts. Treatment for MDR-TB is significantly more expensive and complex than for drug-susceptible TB (DS-TB), with costs reaching up to USD182,000 per patient in some settings (2). Traditional treatment regimens for DS-TB typically last six months; however, recent studies have shown that shorter regimens (e.g., four months) can be equally effective and may reduce costs. For instance, the estimated cost for a 4-month regimen in the United States (US) is around USD23,000 per patient (3). This includes hospitalisation costs, which can account for a significant portion of total expenses (4). The comprehensive management of TB requires extensive healthcare resources, including diagnostic tests (sputum cultures and chest radiographs), hospitalisation for severe cases, and community-based interventions. In Canada, median costs for managing DS-TB were reported at USD12,148 per patient. Hospitalisation alone can contribute up to 61.7% of total costs for isoniazid-resistant TB cases (5). Beyond direct medical costs, the economic impact of TB extends to lost productivity due to illness or death. A study indicated that productivity losses accounted for significant percentages of total costs in various income settings: 16% in high-income countries and up to 40% in low-income countries (4, 6). Given these complexities, there is a pressing need for comprehensive TB management programmes that integrate various interventions. Rapid diagnostic tests such as Xpert MTB/RIF (Mycobacterium tuberculosis/Rifampicin) can significantly enhance detection rates and reduce treatment delays. Active case finding (ACF) and initiatives like mobile health units can improve access to care and facilitate early diagnosis among high-risk populations (7). Patient-centred care models that support adherence can improve treatment outcomes and reduce the risk of developing drug resistance. Understanding and managing the costs associated with drug procurement and treatment delivery is essential for sustainable TB control programmes (1). This systematic review aims to synthesise existing literature on the costs associated with TB treatment programmes, focusing on both direct and indirect expenses related to managing DS-TB and drug-resistant TB.

## Methods

This review used the Preferred Reporting Items for Systematic Reviews and Meta-Analyses (PRISMA) guidelines. It systematically explores the financial implications of a comprehensive TB programme. The eligibility criteria were established using the Population, Intervention, Comparison, Outcomes, and Study Design (PICOS) framework. Specifically, the review focuses on the population of individuals or communities affected by TB, the intervention being the comprehensive TB programme, the outcome being the costs associated with this programme, and the study design being economic evaluations, with no comparison group included. This review protocol is registered at the National Institute for Health Research; International Prospective Register of Systematic Reviews (PROSPERO) with registration number CRD42024585684 at https://www.crd.york.ac.uk/prospero.

### Eligibility Criteria

The studies included in this review adhere to specific inclusion criteria: economic evaluation studies published between 2013 and 2024. Excluded from consideration are observational studies, experimental studies, case-control studies, cohort studies, review articles, and case reports.

### Search Strategy

The articles included in this systematic review were sourced from three online databases: PubMed, Scopus, and ScienceDirect. The Medical Subject Headings (MeSH) terms employed for the systematic search were “costs,” “Tuberculosis,” and “Health Program.” These keywords were used to query each database for relevant articles.

### Study Selection

The studies incorporated in this review adhered to specific criteria, including observational studies. The articles were imported into the Rayyan platform (https://www.rayyan.ai/), where duplicate entries were removed. The investigators independently assessed the titles and abstracts of the publications using predefined criteria to ascertain their inclusion or exclusion. Any disagreements among the reviewers were resolved through discussion. A voting process was employed to reach a final decision if consensus could not be achieved. The inclusion criteria for the studies encompassed preventive and curative programmes, patients with MDR-TB, latent TB, and drug-sensitive TB. Only articles published in English were considered. Articles that were inaccessible or designed explicitly as review studies were excluded from the evaluation.

### Quality Assessment

The number of reviewers involved in this study was six. The quality of all studies included in the analysis was assessed independently using the Critical Appraisal Skills Programme (CASP) for Assessment Risk of Bias of Economic Evaluation Studies (8). Any disagreements were resolved through discussion. The concepts, arguments, and overall intellectual content in this manuscript are entirely the original work of the authors. A generative AI tool named WRITEFULL (https://www.writefull.com/) was used to help authors improve the quality of the manuscript by checking grammar and enhancing readability. Furthermore, the authors also used Rayyan.ai, a basic platform, for the first step of reviewing the manuscript, including checking for duplication and study selection.

### Data Extraction

The data extracted from the eligible studies included author, year, study design, population, intervention, and cost outcomes.

## Results

Two thousand eight hundred thirteen published articles were retrieved from online databases, namely PubMed, Scopus, and ScienceDirect, with publication dates from 2013 to 2024. After removing duplicates, 2,283 articles remained. Upon abstract review, 2,006 articles were excluded due to irrelevance. Two hundred seventy-seven articles were assessed for eligibility, with exclusions: 54 articles due to inappropriate study design, 11 due to inappropriate publication type, 165 due to inappropriate outcomes, 31 due to incorrect population, three due to incorrect study protocol, and two due to study duration. Figure 1 illustrates the PRISMA flowchart detailing the article selection process.

### Bias and Quality of the Included Studies

In this review, the bias of articles was assessed using CASP for economic evaluation studies, as shown in Table 1.

### Characteristics of Included Studies

The characteristics of the studies analysed are summarised in Table 2, which includes details such as the authors, publication years, study designs, populations, interventions, and cost outcomes. These 11 studies were sourced from diverse regions and incorporated into this systematic review. Published between 2013 and 2024, all studies employed an economic evaluation research design and were conducted in institutional or community-based settings. The selected articles specifically address the costs associated with comprehensive TB programmes.

### The Cohort of Patients Participating in the TB Management Programme

Across the 11 included economic evaluation studies, patients enrolled in TB control programmes were generally grouped into two main age categories: 0 to 15 years and older than 15 years, reflecting differences in epidemiological and social risk profiles. Routine case-finding activities, such as contact tracing and age-stratified TB screening, were the predominant entry points for programme enrolment in several studies. Institution- or community-based programmes from Africa, Asia, and other regions consistently reported that these age-specific screening strategies facilitated the systematic identification of eligible TB patients (9–12).

### Strategies Implemented Within the TB Control Programme

Several included studies categorised TB control interventions into three main activities: PCF, ACF, and passive household contact investigation (HCI), which were implemented to strengthen programme effectiveness in African settings. In one study from southern Africa, a TB control strategy combining enhanced contact investigation, routine age-based screening, and an integrated approach was associated with improved case detection and programme outcomes (9).

Evidence from Pakistan demonstrated that systematic contact tracing substantially increased the probability of correctly identifying individuals eligible for TB programme enrolment, with accuracy improving several-fold after the introduction of structured tracing protocols (11). In Georgia, the programme explicitly differentiated between ACF and PCF pathways, which were reported to streamline case identification and improve the efficiency of TB services (13). In Indonesia, an economic evaluation study highlighted that the national TB programme was dominated by comprehensive diagnostic and treatment services, but also emphasised the need for continuous quality improvement in recording systems and treatment outcomes (15). Other studies in this review reinforced the central role of both ACF and PCF as core components of effective TB control strategies.

### Funding the TB Control Initiative

Findings from 11 economic evaluations indicate that funding for TB control programmes is highly heterogeneous, with support from multiple sources, including public budgets and patient out-of-pocket payments. Several studies reported substantial additional patient costs for diagnostic and screening procedures, even for nominally free services. An evaluation from Pakistan reported an average additional expenditure of approximately USD120 per patient for TBrelated care, reflecting the cumulative effect of diagnostic, transport, and ancillary costs (11). A study from Bangladesh documented a marked increase in national expenditure for TB investigation and screening, rising from about USD49.20 million in 2016 to USD146.93 million in 2022 (16).

Economic analyses from several African countries estimated patient-level costs of around USD443.62 per TB case, even within publicly funded programmes (11). For South Africa, one study showed that combining integrated contact investigation with routine screening was associated with an incremental cost-effectiveness ratio of approximately USD9,050 per DALY averted (9). Another analysis reported that a strategy integrating PCF and ACF resulted in an average programme cost of about USD1,492.95 per TB case treated (12). The reported costs of ACF activities ranged from approximately USD16.64 to USD61.08 per person screened, depending on the scale and setting (13).

A separate economic evaluation of MDR-TB management indicated that optimising drug regimens could yield potential savings of approximately USD3 million over the study period (18). Across studies, the estimated funding gap for TB activities reached USD301.62 million between 2018 and 2022, with treatment costs accounting for the largest share of total expenditure (16). In Indonesia, despite the government’s provision of free diagnostic and treatment services, patients were still reported to incur out-of-pocket expenses equivalent to up to 20% of their annual income, highlighting substantial financial hardship (15). In China, increased outpatient visits for TB care were associated with a higher risk of catastrophic health spending, further illustrating the economic vulnerability of TB-affected households (14).

## Discussion

Since its identification in 1882, TB has remained a prevalent global health concern. According to the WHO’s 2024 report, the recorded cases reached 8,456,395, with approximately 9% occurring in individuals aged 0 to 14 (20). The high incidence of TB worldwide has prompted governments to implement various health programmes as preventive and control measures. These initiatives include short message service (SMS) reminders, direct supervision by family members, and directly observed therapy conducted by community health care workers (21).

### The Cohort of Patients Participating in the TB Management Programme

The findings from the systematic review highlight critical insights into the demographics and characteristics of patients participating in TB control programmes, emphasising the importance of age stratification and targeted interventions. Dividing patients into two distinct age categories, those aged 0 to 15 years and those 15 years and older, reflects the varying epidemiological and social factors influencing TB incidence across age groups. This stratification is crucial because it enables tailored strategies that address the unique needs of each demographic, particularly given the heightened vulnerability of younger populations to TB infections (22). According to the WHO report, in 2024, TB predominantly affected males aged 15 years and older (20). Additionally, the primary reason for including populations aged 0 to 15 years in comprehensive TB programmes is the generally low level of public knowledge. Moreover, children aged 0 to 2 years are highly vulnerable to infections, making TB more prevalent among infants. In contrast, the high incidence of TB in individuals aged 15 years and older is primarily attributed to limited awareness, smoking behaviour, alcohol consumption, and exposure to polluted air (23).

Research conducted in Indonesia indicates that social determinants influence the population enrolled in TB control programmes. As identified in the study, these determinants are directly associated with TB incidence and include low levels of knowledge, limited social and community support, and high population density, which is also considered a geographical factor (24). A study conducted in Indonesia also supports the finding that the high number of TB cases is influenced by geographical factors, particularly population density (25). This underscores the need for targeted interventions in high-density areas, where social and environmental factors exacerbate the risk of TB spread (26).

### Strategies Implemented Within the TB Control Programme

The systematic review highlights the diverse interventions employed in TB control programmes, emphasising the categorisation of activities into PCF, ACF, and passive HCI. These distinct strategies are critical for enhancing the effectiveness of TB control efforts, particularly in regions with a high TB burden. The findings from various studies underscore the importance of tailored approaches to case detection and management, essential for improving patient outcomes and reducing transmission rates. Identifying three primary activities in Africa, PCF, ACF, and HCI, demonstrates a comprehensive approach to TB control. When implemented effectively, research indicates that these strategies can significantly improve case detection rates. For instance, a study by Jayasooriya et al. (27) found that systematic active screening can uncover cases that might otherwise remain undiagnosed, thereby contributing to the overall success of TB control programmes. The effectiveness of contact tracing in enhancing TB programme enrolment is particularly noteworthy. The research conducted in Pakistan, which demonstrated a significant increase in the accuracy of identifying individuals for the TB programme through contact tracing efforts, underscores the value of targeted investigations in improving programme outcomes (28). This finding aligns with global best practices in TB control, where proactive case-finding strategies are essential for early detection and treatment initiation, ultimately reducing transmission rates (29). In Georgia, the differentiation between ACF and PCF strategies further illustrates the importance of structured approaches in TB control programmes.

By categorising these strategies, health authorities can optimise resource allocation and enhance the efficiency of case detection processes (30). This emphasises the need for comprehensive and systematic approaches to TB case-finding to improve overall programme effectiveness (31). This aligns with the WHO’s End TB Strategy, which advocates for community-based ACF as a central component of effective TB management (1, 32). The effectiveness of TB control programmes in southern Africa is closely linked to enhanced contact investigation and routine screening based on age demographics. These findings are supported by Saunders et al. (33), who found that ACF strategies increased community members’ awareness of TB symptoms and improved access to testing and treatment. This suggests that educational components integrated into ACF can enhance the overall effectiveness of TB control efforts. Conversely, the findings from Indonesia indicate a focus on providing comprehensive diagnostic and treatment services, yet they also highlight the need for ongoing evaluation and innovation within TB control programmes. The literature echoes the need for continuous improvement in case recording and treatment outcomes, emphasising that effective TB control requires robust diagnostic capabilities and the ability to adapt and refine strategies based on emerging data (34). This is particularly relevant in resource-limited settings where healthcare infrastructure may be inadequate. The WHO’s national TB control guidelines, which include the provision of the Bacille Calmette-Guérin (BCG) vaccination to children and comprehensive TB infection control programmes, further support the need for multifaceted approaches to TB management (35). The survey’s findings align with these guidelines, reinforcing the importance of integrating vaccination, education, and community engagement into TB control strategies (36). In Rome, implementing community-based assistants and training healthcare workers exemplifies a successful strategy that can be replicated in other regions. The European Centre for Disease Prevention and Control (ECDC) emphasises collaboration between health ministries and healthcare services to enhance TB control efforts (37, 38). Such collaborative approaches can foster stronger health systems capable of addressing the complexities of TB management. The systematic review also highlights the critical role of screening and investigation techniques in identifying new TB cases. Research conducted in the US supports the notion that active TB screening significantly improves TB control efforts, particularly in high-risk populations (39, 40). Brooks et al. (41) stated that further emphasise that ineffective screening is often linked to the failure of TB control initiatives, particularly among vulnerable groups such as children. This underscores the need for targeted screening strategies prioritising high-risk populations to ensure timely diagnosis and treatment.

### Funding the TB Control Initiative

The economic dimensions of TB control programmes involve a complex interplay of factors that significantly influence both patient outcomes and overall programme efficacy. Evidence indicates that although some TB-related services may be advertised as free, hidden and out-of-pocket costs impose a substantial financial burden on patients. This complexity is evident across global contexts, where the economic implications of TB management can deter effective care-seeking behaviour and lead to poorer health outcomes.

Research conducted in Pakistan has demonstrated that patients incur an additional expenditure averaging USD120 per patient, reflecting broader trends observed across numerous low- and middle-income countries. Supporting this narrative, studies in several African nations have shown that patients reported incurring costs of approximately USD443.62 per case, underscoring the troubling reality that even government-provided services entail hidden costs that patients must navigate. Out-of-pocket expenses include direct clinical costs, transportation, diagnostics, and ancillary care, which can significantly affect low-income households (19, 42). This scenario is further exemplified by research from Indonesia, where patients incur personal expenses averaging 20% of their annual income despite the availability of free diagnostic and treatment services. Such financial liabilities can severely restrict access to care, influencing individuals’ decisions regarding treatment uptake and adherence (4, 17).

The discrepancy between the official framing of “free” TB services and the financial reality experienced by patients necessitates a nuanced understanding of funding mechanisms within TB control programmes. While governmental bodies may commit to covering treatment costs, the actual economic burden often extends beyond these commitments, compelling patients to shoulder additional expenses. This indicates an urgent need for comprehensive financial planning and robust support mechanisms, including subsidised diagnostic services or financial risk protection strategies explicitly aimed at vulnerable populations (43, 44). By acknowledging and addressing these out-of-pocket costs, healthcare systems can improve access to care and enhance programme effectiveness. Thus, it might impact the health outcome of the society, such as the DALYs saved, and also the cost of the TB programme, more effectively.

Moreover, the consequences of inadequate financial support systems are profound; they reverberate throughout households and communities. Catastrophic health expenditures can culminate in a downward spiral in household income, with families losing productive members due to the combined burden of illness and financial distress (45). In high-burden TB countries such as South Africa and India, this phenomenon exacerbates existing economic inequalities and undermines the feasibility of sustaining long-term treatment plans (46, 47).

The financial implications of TB control extend to treatment modalities, as highlighted by studies comparing the cost-effectiveness of various intervention strategies (48, 49). Given that many TB patients are already economically disadvantaged, treatment choices should not solely depend on clinical efficacy; the economic implications for patients must also be taken into account. Therefore, ensuring equitable access to low-cost or subsidised diagnostic techniques, as well as cost-effective treatment regimens, is crucial to enhancing patient experience and overall health system performance. At the policy level, recognising out-of-pocket costs as a critical barrier to TB care necessitates concerted action to revise existing financial strategies (50). Interventions must integrate financial considerations into TB control frameworks to mitigate economic strain on patients while ensuring treatment adherence. Approaches could include innovative financing mechanisms, such as conditional cash transfers linked to treatment adherence or targeted subsidies that specifically address costs associated with diagnostics and treatment (4, 43).

## Conclusion

This systematic review underscores the pressing need for comprehensive and financially sustainable TB management programmes that consider both direct and indirect costs associated with TB treatment. The findings reveal that MDR-TB carries exorbitant treatment costs that can deter patients from seeking necessary care, thereby perpetuating transmission and negative health outcomes. Moreover, the economic impact extends beyond medical expenses, incorporating productivity losses that disproportionately affect low- and middle-income populations. Effective interventions, such as targeted ACF and patient-centred care models, have proven successful in enhancing treatment adherence and improving health outcomes. Aligning these strategies with the global objective of reducing TB incidence, as outlined in WHO’s End TB Strategy, requires innovative financial mechanisms to address the hidden costs faced by patients. The research emphasises the importance of funding frameworks that alleviate economic barriers and ensure equitable access to high-quality TB care. Thus, developing robust costing models and integrating them into national health policies will be crucial in optimising resource allocation and sustaining long-term improvements in TB control efforts. By addressing these multifaceted economic challenges, health systems can enhance patient access and treatment adherence and, ultimately, reduce the burden of TB worldwide.

## Figures and Tables

**Figure 1:**
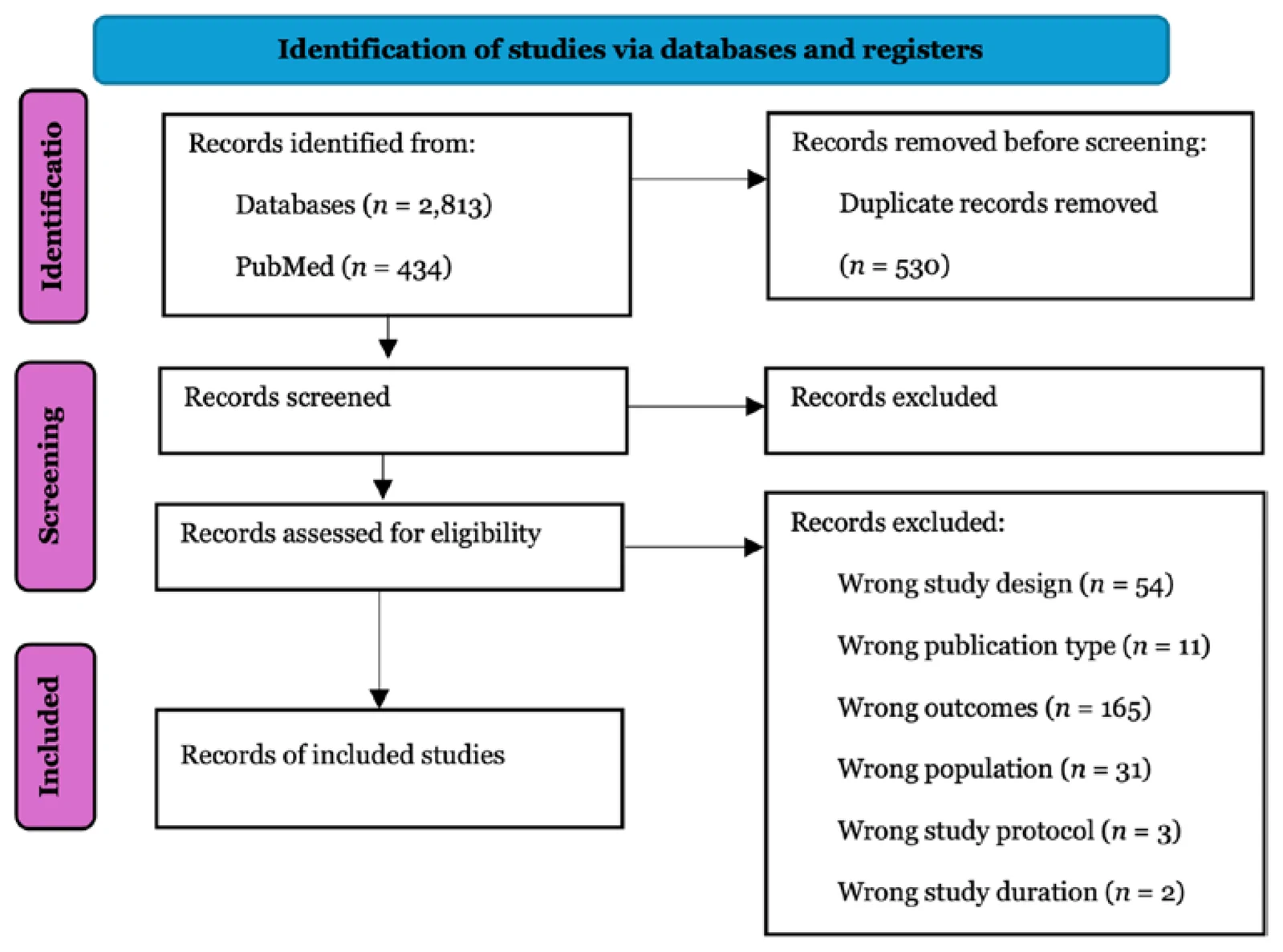
Flow diagram of the research procedure

**Table 1:** CASP critical appraisal tool for assessment of risk of bias for economic evaluation

Authors	Q1	Q2	Q3	Q4	Q5	Q6	Q7	Q8	Q9	Q10	Q11	Q12
Brough et al. (9)	Y	Y	Y	Y	Y	Y	Y	Y	Y	Y	Y	Y
Gomez et al. (10)	Y	Y	Y	Y	Y	Y	Y	Y	Y	Y	Y	Y
Hussain et al. (11)	Y	Y	Y	Y	Y	Y	Y	Y	Y	Y	Y	Y
Sekandi et al. (12)	Y	Y	Y	Y	Y	Y	Y	Y	Y	Y	Y	Y
Chikovani et al. (13)	Y	Y	Y	Y	Y	Y	Y	Y	Y	Y	Y	Y
Jiang et al. (14)	Y	Y	Y	Y	Y	Y	Y	Y	Y	Y	Y	Y
McAllister et al. (15)	Y	Y	Y	Y	Y	Y	Y	Y	Y	Y	Y	Y
Hasan et al. (16)	Y	Y	Y	Y	Y	Y	Y	Y	Y	Y	Y	Y
Menzies et al. (17)	Y	Y	Y	Y	Y	Y	Y	Y	Y	Y	Y	Y
Li et al. (18)	Y	Y	Y	Y	Y	Y	Y	Y	Y	Y	Y	Y
Getahun et al. (19)	Y	Y	Y	Y	Y	Y	Y	Y	Y	Y	Y	Y

Y = Yes, N = No, and C = Cannot Tell

**Table 2:** Summary of the included studies

Author(s) Year Study design	Population	Intervention	Primary outcome
Brough et al. (9) 2023 Economic evaluation	People aged 0 to 15 years old located in South Africa	Three strategies for ACF at the population level were assessed: Enhanced contact investigations for individuals aged 0 to 15 years old Routine screening based on age A combination of enhanced contact investigations and age-based routine screening	The combined strategy (screening, contact tracing, and preventive treatment) was cost-effective at USD9,050 per DALY (95% Confidence Interval: 2,890; 22,920) It remained cost-effective, with an infection risk above 1.6% Routine screening at age 2 was optimal for infection risks between 0.8% and 1.6%
Gomez et al. (10) 2021 Economic evaluation	Primary population: XDR-TB patients Secondary scenario: includes MDR-TB patients with treatment failure/intolerance (FDA/EMA-approved) XDR-TB: MDR-TB resistance + fluoroquinolone and ≥ one injectable (capreomycin, kanamycin, and amikacin) MDR-TB: resistance to isoniazid and rifampicin Treatment failure: no sputum conversion after 6-month intensive phase Intolerance: inability to continue treatment due to adverse reactions to key drugs (e.g., para-aminosalicylic acid and ethionamide)	The intervention is a shortened, all-oral regimen for XDR-TB, including BPaL, lasting 6 months The duration may extend to 9 months if sputum conversion does not occur after 4 months of treatment	Cost-saving potential: BPaL for XDR-TB is cost-saving at global drug facilities’ pretomanid price across all settings, tied to XDR-TB prevalence 5-year savings: ≈USD3 million (South Africa), ≈USD200,000 (Georgia), and ≈USD60,000 (Philippines) South Africa: future antiretroviral therapy costs reduce savings to ≈USD1 million Expanded use: including MDR-TB treatment failures/intolerance enhances savings and clinical impact Threshold prices: USD500 (South Africa, with antiretroviral therapy) vs. USD3,650 (Georgia) and USD3,800 (Philippines) for cost-neutrality
Hussain et al. (11) 2021 Economic evaluation	All children from Sindh, Pakistan, visiting these facilities were verbally screened for TB symptoms Children deemed at high risk for TB underwent further investigation A total of 176 children were selected as participants in this study	Passive contact investigation involves evaluating households with members infected with TB A limitation is that household contacts screened were recorded only as TB contacts, not linked to a specific index TB patient There is no routine follow-up to verify if contacts attended the facility for screening The article does not specify the intervention period	Enhanced contact investigation detected 3.8 times more TB cases per index patient than passive contact investigation alone The incremental cost for enhanced contact investigation was USD30 per index patient, resulting in USD120 per additional TB case identified Active contact investigation was 1.5 times more effective than enhanced contact investigation The incremental cost for active contact investigation was USD238 per additional TB case identified
Sekandi et al. (12) 2015 Economic evaluation	Urban population aged more than 15 years old	PCF alone (> 2 weeks) PCF + ACF (> 2 weeks) PCF + HCI	The PCF + HCI strategy was cost-effective at USD443.62 per additional TB case detected, outperforming PCF alone PCF+ACF was not cost-effective at USD1492.95 per additional TB case detected Sensitivity analyses showed PCF + ACF could become cost-effective if: Chronic cough prevalence in the ACF population rose from 4% to 40% ACF programme costs were cut by 50%
Chikovani et al. (13) 2021 Economic evaluation	The population is sourced from Georgian healthcare facilities These facilities provide ACF/PCF, diagnostic tests, and outpatient and inpatient TB services Some facilities offer multiple services Total sites: *n* = 133	ACF and PCF Diagnostic tests Outpatient services for TB Inpatient services for TB No specific period	The cost for ACF: iFAST (an additional TB case detected and initiated on treatment): USD61.08 Contact visits: USD18.36 Contact tracing epidemiologists: USD16.64 The cost for PCF: Adult PTB: USD226.13 Adult EPTB: USD10.67 Child PTB: USD221.22 The cost of TB prevention: Children under 5 years (contact - 3 HR [3 months of daily isoniazid plus rifampicin]): USD9.99 Children under 5 years (contact - HIV 6 H [6 months of daily isoniazid treatment]): USD15.60
Jiang et al. (14) 2019 Economic evaluation	TB patients from Yichang City, Zhenjiang, and Hanzong. The population was 540 patients	The usual treatment applies to 270 patients, and 270 patients have terminal stages (no specific treatment for some time)	The study included 530 patients Inpatient rates significantly increased from 33.5% to 75.9% (*P* < 0.001), with the most significant increase among low-income patients Outpatient visits rose from 4.6 to 5.6 (*P* < 0.001), with the most notable increase among the poorest patients Compared to developed countries, the increase in outpatient visits for illness was more significant in the remote Wufeng county
McAllister et al. (15) 2020 Economic evaluation	Adults with TB from community health centres (CHCs), public and private hospitals, and private practitioners in Bandung City, West Java	The interview gathered demographic data such as age, gender, education, and ethnicity Clinical information included diagnosis date, treatment details, and TB history Cost data was also collected An adapted questionnaire from WHO’s “Tool to Estimate Patient Costs” for Indonesia was used It addressed patient and health system delays, direct and indirect costs, coping costs, and socioeconomic factors	For 26.5% of patients, total costs represented 20.0% of their annual household income Despite free TB diagnostic and treatment services in Bandung, patients faced substantial out-of-pocket expenses Increasing National Health Insurance enrolment and improving early TB detection will help lower these costs
Hasan et al. (16) 2023 Economic evaluation	Facility-related data were collected from 12 purposively selected facilities These facilities represent various levels of diagnosis and treatment Both government and nongovernmental organisation service providers were included	Conducted consultative meetings with experts and officials/managers Reviewed documents and databases Visited five purposively selected TB healthcare facilities Compared estimated costs with funds allocated to the NTP from 2018 to 2022 to assess the funding gap	A funding gap was identified for the NTP from 2018 to 2022 The estimated NTP cost was USD49.22 million in 2016, projected to increase to USD146.93 million by 2022 Significant expenses included human resources (41.1%) and medicines/supplies (38.0%) The highest treatment cost was for XDR-TB, at USD7,422.4 in 2016 From 2018 to 2022, NTP costs are expected to total USD536.8 million, exceeding the current allocation by USD235.18 million
Menzies et al. (17) 2016 Economic evaluation	Documents from national TB programmes in selected countries	Intervention scenarios were created with country stakeholders to enhance existing interventions for high coverage by 2025 Nine modelling groups estimated policy outcomes and costs using service use estimates, cost data, and expert implementation opinions Health effects (DALY saved) and resource implications for 2016–2035, including patient costs, were evaluated Scenarios were compared to a base case of current practices to assess resource needs and cost-effectiveness	Intervention scenarios were developed with stakeholders to scale up coverage by 2025 Policy outcomes, costs, health effects (DALYs saved), and resource needs for 2016–2035 were estimated by nine modelling groups. Scenarios were compared to current practices to evaluate cost-effectiveness and resource requirements
Li et al. (18) 2015 Economic evaluation	All patients with smear-positive pulmonary TB diagnosed in Centre for Disease Prevention and Control clinics and hospitals were tested for MDR-TB Testing was conducted using molecular and conventional drug susceptibility tests The programme was implemented in four medium-sized, third-tier cities in China, selected based on economic development The cities are Hohhot, Kaifeng, Lianyungang, and Yongcuan	They compared data from a 12-month programme period (2011) to those from a retrospective survey of all patients with MDR-TB diagnosed in the same cities during a baseline period (2006–2009)	The comprehensive programme significantly improved access to diagnosis and quality treatment for MDR-TB It made treatment more affordable The programme could facilitate China’s achievement of universal access to MDR-TB care
Getahun et al. (19) 2016 Economic evaluation	Adult TB patients who were directly observed during treatment in 27 public health facilities in Addis Ababa, Ethiopia	Interviewer-administered questionnaire adapted from the Tool to Estimate Patients’ Costs	Despite free anti-TB drugs, patients incur catastrophic out-of-pocket expenses These financial burdens impede TB eradication efforts Patients in resource-limited countries require integrated, patient-centred care Comprehensive health insurance, financial incentives, and nutritional support are crucial to reduce these costs

XDR-TB = extensively drug-resistant tuberculosis; BPaL = Bedaquiline, Pretomanid, Linezolid; PTB = pulmonary tuberculosis; PCF = passive case finding; DALY = disability-adjusted life year; ACF = active case finding; MDR-TB = multidrug-resistant tuberculosis; TB = tuberculosis; HCI = household contact investigation; EPTB = extrapulmonary TB; NTP = national tuberculosis program
